# RhoA signaling modulates cyclin D1 expression in human lung fibroblasts; implications for idiopathic pulmonary fibrosis

**DOI:** 10.1186/1465-9921-7-88

**Published:** 2006-06-15

**Authors:** KL Watts, E Cottrell, PR Hoban, MA Spiteri

**Affiliations:** 1 Lung Research, Institute of Science and Technology in Medicine, University Hospital of North Staffordshire/Keele University, Staffordshire, UK

## Abstract

**Background:**

Idiopathic Pulmonary Fibrosis (IPF) is a debilitating disease characterized by exaggerated extracellular matrix deposition and aggressive lung structural remodeling. Disease pathogenesis is driven by fibroblastic foci formation, consequent on growth factor overexpression and myofibroblast proliferation. We have previously shown that both CTGF overexpression and myofibroblast formation in IPF cell lines are dependent on RhoA signaling. As RhoA-mediated regulation is also involved in cell cycle progression, we hypothesise that this pathway is key to lung fibroblast turnover through modulation of cyclin D1 kinetic expression.

**Methods:**

Cyclin D1 expression was compared in primary IPF patient-derived fibroblasts and equivalent normal control cells. Quantitative real time PCR was employed to examine relative expression levels of cyclin D1 mRNA; protein expression was confirmed by western blotting. Effects of Rho signaling were investigated using transient transfection of constitutively active and dominant negative RhoA constructs as well as pharmacological inhibitors. Cellular proliferation of lung fibroblasts was determined by BrdU incorporation ELISA. To further explore RhoA regulation of cyclin D1 in lung fibroblasts and associated cell cycle progression, an established Rho inhibitor, Simvastatin, was incorporated in our studies.

**Results:**

Cyclin D1 expression was upregulated in IPF compared to normal lung fibroblasts under exponential growth conditions (p < 0.05). Serum deprivation inhibited cyclin D1 expression, which was restored following treatment with fibrogenic growth factors (TGF-β1 and CTGF). RhoA inhibition, using a dominant negative mutant and a pharmacological inhibitor (C3 exotoxin), suppressed levels of cyclin D1 mRNA and protein in IPF fibroblasts, with significant abrogation of cell turnover (p < 0.05). Furthermore, Simvastatin dose-dependently inhibited fibroblast cyclin D1 gene and protein expression, inducing G1 cell cycle arrest. Similar trends were observed in control experiments using normal lung fibroblasts, though exhibited responses were lower in magnitude.

**Conclusion:**

These findings report for the first time that cyclin D1 expression is deregulated in IPF through a RhoA dependent mechanism that influences lung fibroblast proliferation. This potentially unravels new molecular targets for future anti-IPF strategies; accordingly, Simvastatin inhibition of Rho-mediated cyclin D1 expression in IPF fibroblasts merits further exploitation.

## Background

Idiopathic pulmonary fibrosis (IPF) is an insidious fibroproliferative disorder, characterised by interstitial alveolar fibrosis thought to be consequent on aberrant responses to undefined microinsults. Lung injury maybe exacerbated by concurrent failure of re-epithelialisation and excessive fibroblast differentiation [1,2], underpinned by erratic deposition of extracellular matrix (ECM) proteins and progressive lung tissue remodelling. Although a number of scientific advances have been made in understanding disease pathogenesis, no efficacious therapy is available to halt or alter these exaggerated pro-fibrotic processes.

It follows that IPF pathogenesis must involve aberrations within regulatory pathways critical to the described cellular – biomolecular events. Under such conditions, fibroblasts acquire an aggressive, contractile myofibroblast phenotype, with potent capability for ECM protein production [3]. Fibroblast-myofibroblast differentiation, is driven by an upregulated pool of growth factors, of which connective tissue growth factor (CTGF) is a key player [4]. CTGF induction primarily, but not exclusively, is mediated by TGF-β1 through a TGF-β response element in the CTGF promoter [5]. CTGF modulates IPF fibroblast differentiation through a signalling pathway involving RhoA [6,7]. Interestingly, RhoA is also known to be instrumental in the kinetics of cyclin D1 expression, specifically in G1 phase of the cell cycle [8]. It follows that as relentless proliferation and differentiation of fibroblasts are crucial to IPF progression, deregulated expression of key cell cycle genes and transcription factors may be pivotal to disease pathogenesis.

The cell cycle regulator cyclin D1 is a critical factor in the development of proliferative disease [9], including specific organ oncogenesis [10-12]. This 36-kDa protein has a widely accepted role in positive regulation of G1-S progression [13]. Functioning as a 'mitogenic sensor', in the presence of growth factors, cyclin D1 gene (*CCND1*) drives target cells through the restriction point in the G1 phase of their cycle (thus committing them to cell division). This function is facilitated through binding and activation of cyclin-dependent kinases (CDK) 4 and 6, with phosphorylation of the retinoblastoma protein (Rb), and release of sequestered transcription factors such as E2F [14,15]. Furthermore, *in vitro *induction of *CCND1 *augments cellular proliferation and transformation of mammalian cells [16]; which in rodent cells is characterised by a shortened G1 phase with reduced dependence on mitogens [17].

A key histological feature of IPF lungs is presence of fibroblast proliferation, with fibroblastic foci formation. We hypothesise that cyclin D1 plays an instrumental role in these pro-fibrogenic processes, augmented by *in situ *growth factor overproduction and exaggerated extracellular matrix deposition [18]. We contend that cyclin D1 influence in fibroblasts is mediated via a RhoA signalling pathway, especially as RhoA is known to regulate G1 progression of cells [19]. Accordingly, our study explores for the first time expression levels of cyclin D1 in IPF patient-derived fibroblasts (and equivalent controls) and identifies the influence of Rho, using constitutively active and dominant negative RhoA constructs as well as pharmacological inhibitors, including the agent Simvastatin. This agent selectively blocks a key cascade enzyme, 3-hydroxy-3-methylglutaryl coenzyme A reductase (HMG CoA), inhibiting essential post-translational modification of RhoA, thus inactivating its signalling function.

## Methods

### Human lung fibroblast cell culture

Three separate human lung fibroblast cell lines isolated from IPF patients (LL29 and LL97a both ATCC, Manassas, USA; and HIPF – a generous gift from R.J. McAnulty, UCL London,) and normal control equivalents (CCD8LU, ATCC, Manassas, USA). The control cell line (CCD8LU) is an adult lung fibroblast cell line, derived from a 48 year old male with cerebral thrombosis, which are a good representative control cell line for analysis of IPF specific effects. All cells were cultured in Dulbecco's modified Eagles medium (DMEM, Sigma Aldrich, Dorset, UK). Media was supplemented with penicillin/streptomycin (100 U/ml) and L-glutamine (2 mM) (both Gibco BRL, Paisley, Scotland) with 10% fetal calf serum (FCS, Labtech, Sussex, UK). All cell lines were cultured and utilized at passages 5–8 to limit passage dependent effects on the observed effects. For experiments, medium was replaced with serum free DMEM (SF-DMEM), for 48 hours to induce quiescence before treatment.

### Treatment with fibrogenic growth factors

Following serum depravation for 48 hours the fibroblasts were stimulated with fibrogenic growth factors; human recombinant TGF-β1 (R&D systems, Oxford, UK) dose of 1 ng/ml and 5 ng/ml; and human recombinant CTGF (Fibrogen, CA, USA) doses of 10 ng/ml and 100 ng/ml. Fibroblasts were treated with the above-mentioned growth factors for 8 hours for gene expression analysis and 24 hours for protein expression studies. The chosen time points and concentrations of growth factors were determined and established in previous and ongoing studies within our laboratories [6,7].

### C3 exotoxin treatment of lung fibroblasts

Quiescent lung fibroblasts were incubated overnight (16 hours) with *Clostridium botulinum *C3 exotoxin (Upstate cell signalling solutions, NY, USA) in SF-DMEM. C3 exotoxin was used at concentrations of 0.5 μg/ml, 1 μg/ml and 5 μg/ml; these doses have been previously shown to inhibit Rho signalling pathways in similar fibroblast lines [6].

### Simvastatin treatment

Simvastatin is used clinically for the treatment of hypercholesterolaemia due its ability to abrogate the cholesterol synthesis pathway via HMG CoA inhibition. The statins also possess a range of secondary effects arising from disruption of guanosine triphosphatase (GTPase) signalling, including members of the Rho and Ras family. Simvastatin (Merck Sharp and Dohme, Hertfordshire, UK) was dissolved and filter sterilised before use in cell culture studies [20]. Quiescent lung fibroblasts were then incubated with physiological concentrations of Simvastatin (0.1 μM, 1 μM 10 μM) for 16 hours in serum free cell culture media. Following Simvastatin pre-conditioning, cells were stimulated with human recombinant TGF-β1 (R&D systems, Oxford, UK) at a dose of 5 ng/ml, cells were harvested at 8 hours for mRNA studies and 24 hours for protein analysis.

### Transient transfection of dominant negative/constitutively active RhoA constructs

Transfection of dominant-negative and constitutively active RhoA (accession number L25080) constructs into human lung fibroblasts (IPF-derived and CCD8LU cells) were performed using Transfast mammalian transfection system (Promega, Southampton, UK). Transfection was performed in lung fibroblasts at 90% confluency following the manufacturer's recommendations. 0.75 μg of DNA was transfected per well (18 mm diameter) using a 1:1 ratio of DNA/Transfast reagent in serum-negative cultures. 90% confluent cells were incubated in the transfection mix containing the RhoA plasmid for 1 hour; DMEM containing 10% FCS was added up to a volume of 1 ml, and cultures were left for 4 hours. Following this, the transfected cells were serum deprived for 48 hours before treatment with TGF-β1 (5 ng/ml) for 8 hours. RhoA G14V (a construct containing a mutation at G14V to render it constitutively active) and RhoA T19N (a construct containing a mutation at T19N, giving it a dominant negative phenotype) constructs were utilized in a cDNA3.1+ vector and were obtained from the Guthrie research institute .

### Real time PCR

Stored cDNA samples isolated from normal and IPF isolated lung fibroblasts were used to assess CTGF and α-SMA gene expression. 2 μl of undiluted cDNA was used per 25 μl reaction; the primer and probe sets were 'pre-designed assay on demand' probes (Applied Biosystems, Foster City, CA); these pre-designed primers are tested and standardised for reproducible expression analysis. Primer and cDNA were added to the TaqMan universal PCR master mix (Applied Biosystems, Foster City, CA), containing all the reagents for PCR. Absolute quantification of the PCR products was carried out with an ABI prism 7000 (Applied Biosystems, Foster City, CA) utilising the relative standard curve method. cDNA that positively expresses the target gene is used to create a dilution series with arbitrary units. To ensure reproducibility, quantitative data were taken at a point in which each sample was in the exponential phase of amplification. The mean quantity of target gene expression was determined from the generated standard curve; then all samples were normalised against an internal standard β actin or 18s in all quantitative PCR reactions. All data are presented as the fold-change over control in cyclin-D1 gene expression.

### Western blotting

Total cell proteins were extracted in lysis buffer comprising 1% (v/v) Triton X-100, 20 mM Tris HCL (pH 8.0), 10% (v/v) glycerol, 1 mM sodium orthovanadate, 2 mM EDTA, 1 mM phenylmethylsulfonyl fluoride (PMSF), 20 μM leupeptin and 0.15 U/ml aprotinin. Recovered cells were lysed in above lysis buffer and placed on ice for 20 minutes. The lysates were then centrifuged at 10 000 g, 4°C to pellet cell debris. The supernatant containing the protein was recovered and assayed for total protein using a commercial microplate assay (Bio-Rad, Hemel Hempsted, UK). 25 μg of total protein was combined with sample buffer and boiled prior to gel loading. In addition full-length, recombinant human cyclin D1 protein a 61 Kda tagged fusion protein corresponding to amino acids 1–295 (Santa Cruz Biotechnology, CA, USA) was also loaded onto the gels to ensure detection of the protein of interest. Proteins were resolved on a 12.5% polyacrylamide gel by electrophoresis at 120 V in reducing buffer and transfer was carried out at 100 V. Membranes were blocked with 5% (w/v) BSA in TBS-T buffer overnight. For detection of the cyclin D1 protein DCS-6 (Santa Cruz Biotechnology, CA, USA) antibody was used at 1:100 dilution in TBS-T and 1% BSA. Secondary detection was carried out with horseradish peroxidase-conjugated (HRP) Affinipure goat anti-mouse IgG antibody (Jackson Immunoresearch) at 1:25,000 in TBS-T containing 1% BSA. The cyclin D1 band was visualised by enhanced chemiluminescence (ECL; Amersham Pharmacia Biotech, Buckinghamshire, UK) according to the manufacturer's recommendations and blots were quantified by densitometrical analysis, which involved correcting each blot for background density on each gel. Ponceau S staining of blots after transfer revealed equal loading of total protein; additionally the membranes were reprobed for GAPDH using rabbit polyclonal antibody to GAPDH (1:1000 dilution, Abcam, UK) to ensure equal loading.

### DNA synthesis of proliferating cells

DNA synthesis was assessed by colorimetric cell proliferation Biotrak ELISA method according to the manufacturer's recommendations (Amersham Biosciences, UK) based on the measurement of 5-bromo-2'-deoxyuridine (BrdU) incorporation during DNA synthesis of proliferating cells. Briefly 30,000 cells were seeded per well of a 96 well plate and left for 24 hours. Cells were then synchronised *in situ *by incubation with serum-depleted media for 48 hours. Cells were then treated with the recognised Rho inhibitor *Clostridium botulinum *C3 exotoxin, (0.5–5 μg/ml) (Upstate cell signalling solutions, Lake Placid, NY) overnight prior to treatment with recombinant human TGF-β1 (5 ng/ml) for up to 5 days. BrdU incorporation was measured daily, during which cells were subjected to BrdU incorporation for 4 hours. The colorimetric change was measured at 450 nm on a Dynatech MR50000 microplate reader (Dynex Laboratories, UK).

### FACS analysis

LL97a lung fibroblasts were grown to approximately 60% confluency prior to serum deprivation for 48 hours (this ensures the cells become quiescent and are synchronised in the cell cycle). The lung fibroblasts were then treated, accordingly with Simvastatin (0.1 μg/ml or 10 μg/ml) with or without TGF-β1 (5 ng/ml) for 24 hours. The cells were then harvested and the cell suspension fixed in 70% ice-cold ethanol. The cell suspension was centrifuged at 200 rpm and the cell pellet resuspended in PBS. RNase (1 mg/ml) and propidium iodide (0.5 mg/ml) were added and incubated for 30 minutes at 37°C. To ensure no clumping of the cells the suspension was passed through a 25 g needle. The cells were analysed on a MOFLO cell sorter (Dakocytomation, Glostrup, Denmark) at a wavelength of 488 nm and speed of 100 events per second (eps). A minimum of 20,000 events per data profile was collected.

### Statistical analysis

Data are shown as a mean ± SEM. An unpaired student's t test was employed for comparing 2 groups of data. Multiple comparisons were made using analysis of variance (ANOVA) followed by Tukeys pairwise comparison. All p values < 0.05 were considered significant.

## Results

### Cyclin D1 gene expression is upregulated in IPF fibroblasts

**Figure 1:**
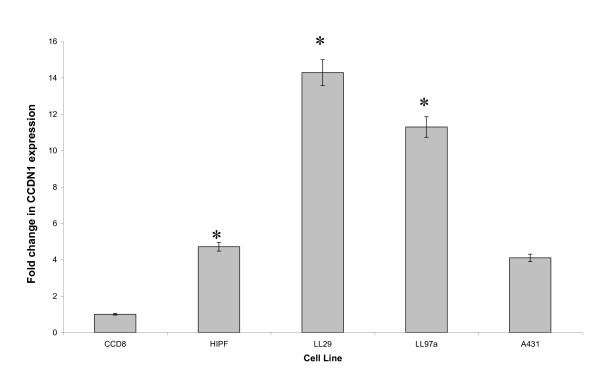
**Expression levels of cyclin D1 mRNA in human lung fibroblasts during exponential growth**. Quantitative real-time RT-PCR was performed on three separate human lung fibroblast cell lines from IPF patients (HIPF, LL29, and LL97a) and normal control equivalents CCD8LU. Quantification of mRNA was performed by determining the threshold cycle; and standard curves were constructed using the values obtained from serially diluted positively expressing human cDNA. All cells were under conditions of exponential growth (10% FCS supplemented media). 3 PCR reactions were performed from 3 independent cell culture experiments, graph represents mean cyclin D1 expression ± S.E.M; * = p < 0.05.

The expression of the cyclin D1 gene was quantified in 3 IPF-derived lung fibroblast cell lines (HIPF, LL29, LL97a) and the adult normal lung fibroblast cell line CCD8LU using a real time PCR approach (Fig 1). Under exponential growth conditions (cells grown in 10% FCS, i.e. actively dividing cells), IPF-derived lung fibroblasts demonstrated a 4.72 to 11.29 fold elevation of cyclin D1 mRNA expression (average of 10.10 fold increase) compared to the CCD8LU normal lung fibroblast cell line (p < 0.05). We compared these data to A431 cells, a human epithelial squamous carcinoma cell line with a known 5 fold amplification of cyclin D1 [21]; the IPF fibroblast cell lines studies significantly exceeded the amplified cyclin D1 mRNA expression of A431 by an average of 2.45 fold.

### Cyclin D1 gene and protein levels are augmented by growth factors

**Figure 2:**
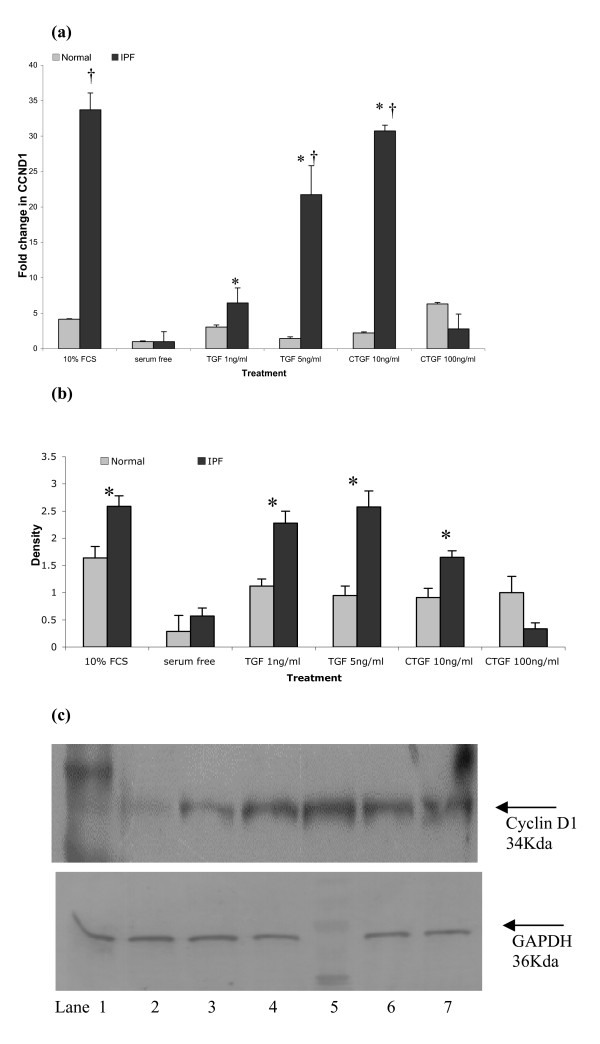
**Expression of cyclin D1 mRNA and protein in human lung fibroblasts; response to fibrogenic growth factors**. Figure 2a: Cyclin D1 mRNA levels were determined by quantitative real-time PCR on three separate human lung fibroblast cell lines from IPF patients (HIPF, LL29, and LL97a) and normal control equivalents CCD8LU exposed to fibrogenic mediators TGF-β1 (5 ng/ml and 10 ng/ml) and CTGF (10 ng/ml and 100 ng/ml) for 8 hrs. Data shown demonstrates analysis from LL97a and CCD8LU fibroblasts, no significant difference was observed in baseline cyclin D1 expression between the cell lines. Data are representative of 3 independent experiments, within each of which PCRs were performed in triplicate. Data represents mean cyclin D1 expression ± S.E.M; * = p < 0.05 compared to serum free, † = p < 0.05 compared to normal lung fibroblasts. Figure 2b: Quantification of cyclin D1 protein expression was performed by western blotting in all three IPF fibroblast lines and normal equivalents. Quiescent serum deprived lung fibroblasts were stimulated with fibrogenic growth factors for 24 hours. Cyclin D1 protein levels found in 25 μg of total protein from normal and IPF derived lung fibroblasts was determined by western blotting. Data shown demonstrates analysis from LL97a and CCD8LU fibroblasts. Data are representative of 3 independent westerns and represented as mean density ± SEM. * = p < 0.05 compared to normal lung fibroblasts. Figure 2c: Representative western blots for cyclin D1 and GAPDH protein expression in a representative IPF lung fibroblast cell line (LL97a). (i) cyclin D1 blot-lane 1 = marker lane 2 = control (serum deprived); lane 3 = 10% FCS; lane 4 = TGF-β1 1 ng/ml; lane 5 = TGF-β1 5 ng/ml; lane 6 = CTGF 10 ng/ml; lane 6 = CTGF 100 ng/ml. (ii) GAPDH blot-lane 1 = control (serum deprived); lane 2 = 10% FCS; lane 3 = TGF-β1 1 ng/ml; lane 4 = TGF-β1 5 ng/ml; lane 5 = marker; lane 6 = CTGF 10 ng/ml; lane 6 = CTGF 100 ng/ml. Ponceau S staining of blots after transfer revealed equivalent loading of total protein.

Cyclin D1 gene expression was measured in the normal lung fibroblasts and the 3 IPF-derived lung fibroblast cell lines following growth factor treatment (Fig 2a), Cells were serum deprived for 48 hours to ensure quiescence and to synchronise cell proliferation; cells were then exposed to physiologically relevant concentrations of growth factors (CTGF and TGF-β1) known to be implicated in IPF pathogenesis [4,5]. Serum deprivation inhibited cyclin D1 expression (as expected); however expression was restored upon treatment with recombinant growth factors. Cyclin D1 augmentation was more pronounced in the IPF-derived lung fibroblasts, especially in the presence of TGF-β1 (1 ng/ml and 5 ng/ml) and CTGF (10 ng/ml) (p < 0.05). Interestingly, in cultures containing the higher concentration of CTGF (100 ng/ml), we observed fibroblast apoptosis especially in IPF-related cell line (data not shown); and no further increase in cyclin D1 expression. It is also of interest to note that in the absence of mitogens the levels of cyclin D1 mRNA are not significantly different between the cell lines studied and expression lies are within are narrow range (2.10–6.65 × 10^-4^).

To further confirm above findings, cyclin D1 protein expression was determined in the same cell lines under the same experimental conditions using western blot analysis (Fig 2b). We observed the same patterns of expression and induction in cyclin D1 protein, reflecting results of cyclin mRNA expression. A representative blot from one of the IPF derived cell lines is shown in Fig 2c. Data shown in fig 2a, 2b and 2c is taken from the patient cell line LL97a; these mRNA and protein data reflect results obtained with the other 2 IPF fibroblast cell lines studied.

### RhoA modulates cyclin D1 gene and protein levels in lung fibroblasts

**Figure 3:**
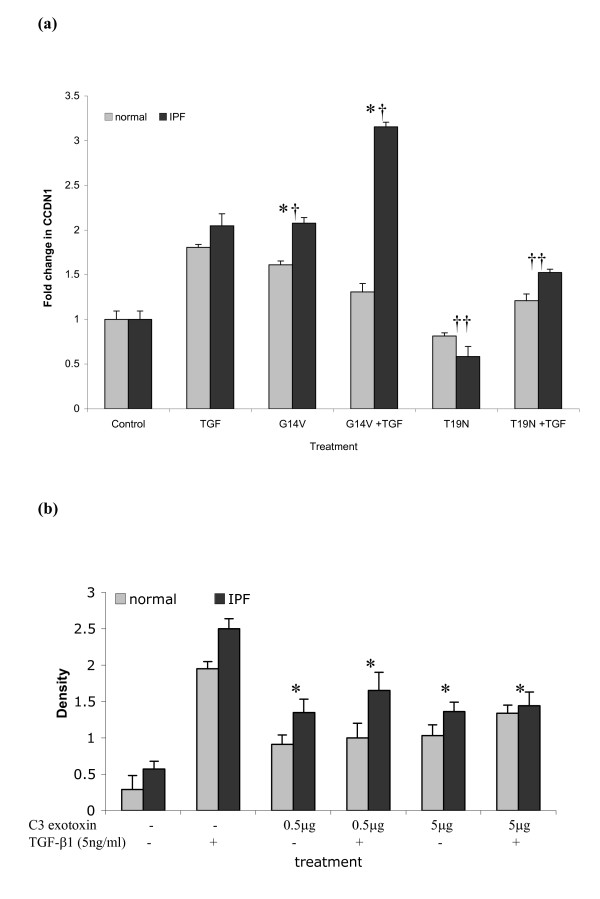
**RhoA signalling directly influences cyclin D1 mRNA and protein expression in lung fibroblasts**. Fig 3a. Human lung fibroblasts (3 IPF fibroblast lines and normal equivalents) were transfected with RhoA T19N (dominant negative) and RhoA G14V (constitutively active) constructs. Cells were serum deprived for 48 hours post transfection before incubation with TGF-β1 (5 ng/ml) for 8 hours. Data shown demonstrates analysis from LL97a and CCD8LU fibroblasts. Data are representative of transfection performed in triplicate from three independent experiments. Data are expressed as mean fold change in cyclin D1 transcript ± SEM. * = p < 0.05 relative to control, † = p < 0.05 relative to normal lung fibroblasts, †† = p < 0.05 relative to TGF-β1 treated fibroblasts. Fig 3b. Human lung fibroblasts (normal CCD8LU and IPF-derived HIPF, LL29 and LL97a) were treated with 0.5 μg/ml and 5 μg/ml of C3 exotoxin with or without subsequent TGF-β1 (5 ng/ml) stimulation. The control shown represents fibroblasts not exposed to C3 exotoxin and/or TGF-β1. Cyclin D1 protein levels found in 25 μg of total protein was determined by western blotting. Data shown demonstrates analysis from LL97a and CCD8LU fibroblasts. Data is representative of westerns performed in triplicate and shown as mean density ± SEM, * = p < 0.05.

Transient transfection of dominant-negative RhoA (RhoA T19N) and constitutively active (RhoA G14V) constructs were utilised to confirm the regulatory role of Rho in cyclin D1 induction (Fig 3a). These data revealed that cyclin D1 mRNA expression levels are of comparable magnitude between cells stimulated with TGF-β1 (5 ng/ml) alone compared to those expressing constitutively active RhoA (G14V RhoA transfected). When G14V active, RhoA cultures were subsequently conditioned with TGF-β1, significant upregulation (p < 0.05) in cyclin D1 gene expression was observed in LL97a; this trend was replicated in the other 2 IPF derived cell lines studied. These data support a role for Rho in cyclin D1 induction; which is further confirmed by the use of a dominant-negative RhoA construct. Transfection with Rho T19N induced significant reduction (p < 0.05) in cyclin D1 gene expression producing a 25.5% and a 33% reduction in normal and IPF derived lung fibroblasts respectively compared to cells treated with 5 ng/ml TGF-β1 alone, further supporting involvement of RhoA in cyclin D1 expression. In our experiments, we did not observe complete inhibition of cyclin D1 gene, as expected of the transient transfection method used. As the average transfection efficiency achieved was about 40%, thus a proportion of the cells in our culture will not have inhibited RhoA signalling. The above result trends were consistent throughout the 3 IPF cell lines analysed.

To confirm above findings, cyclin D1 protein expression was analysed following pharmacological inhibition of RhoA utilising C3 exotoxin (a recognised inhibitor of RhoA) (Fig 3b). Compared to TGF-β1 treatment alone (5 ng/ml), both test concentrations of C3 exotoxin significantly (p < 0.05) abrogated cyclin D1 protein expression in both normal and IPF lung fibroblasts, irrespective of subsequent TGF-β1 exposure.

### DNA synthesis is suppressed by RhoA inhibition

**Figure 4:**
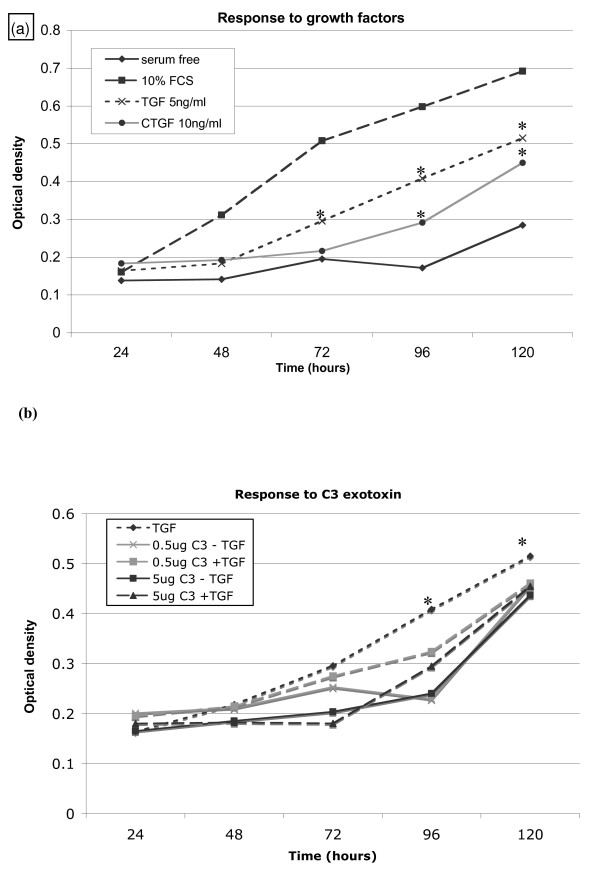
**Cell proliferation is enhanced in IPF lung fibroblasts and can be abrogated by RhoA inhibition**. 4a. Cell proliferation was determined by incorporation of BrdU in three separate human lung fibroblast cell lines from IPF patients (HIPF, LL29, and LL97a) and normal control equivalents CCD8LU data represents analysis in the IPF lung fibroblast cell line LL97a. Cells were subjected to BrdU incorporation at 24-hour intervals as described. Data are representative of the mean of 3 independent experiments (standard error bars have been omitted to simplify the figure). * = p < 0.05 significant elevation relative to serum free controls. 4b. Cell proliferation in response to the recognised Rho inhibitor C3 exotoxin (0.5–5 μg/ml) with or without subsequent TGF-β1 (5 ng/ml) stimulation was measured by BrdU incorporation in three separate human lung fibroblast cell lines from IPF patients (HIPF, LL29, and LL97a) and normal control equivalents CCD8LU. Data shows analysis in the IPF cell line LL97a. Data are representative of the mean of 3 independent experiments (standard error bars have been omitted to simplify the figure). * = p < 0.05 relative to TGF-β1 stimulated cells.

We used a sensitive BrdU incorporation ELISA that measures DNA synthesis to determine if Rho inhibition would alter cell proliferative capability. Firstly DNA synthesis in response to growth factor treatment was determined (Fig 4a). Actively dividing cells (cultured in media supplemented with 10% FCS) had the fastest DNA synthesis rate. As expected, which was almost halted in serum-deprived (quiescent) cells; but exhibited some restoration over the 120-hour time course on exposure to fibrogenic factors TGF-β1 (5 ng/ml) and CTGF (10 ng/ml). At the end time point (120 hr), TGF-β1 and CTGF induced a 44.85% and 36.88% increase respectively (p < 0.05) in BrdU incorporation IPF fibroblasts compared to serum-starved equivalent controls. This data is representative of all 3 IPF cell lines studied. Similar trends are replicated in the normal fibroblast equivalents although the magnitude of BrdU incorporation was approximately 3-fold lower in the controls (data not shown).

Involvement of RhoA in above DNA synthesis was determined using C3 exotoxin, which specifically ADP ribosylates and inactivates Rho. The inhibitor was used at optimal concentrations of 0.5 μg/ml and 5 μg/ml with or without additional TGF-β1 (5 ng/ml) stimulation (Fig 4b). Exposure to C3 exotoxin inhibited BrdU incorporation, even in the presence of TGF-β1 at both 0.5 and 5 μg/ml concentrations. Both C3 exotoxin treatments suppressed DNA synthesis over the 120 hour time course compared to control IPF lung fibroblasts treated with 5 ng/ml TGF-β1 alone; becoming significant at p < 0.05 over time from 72 hours onwards.

### Simvastatin inhibits fibroblast cyclin D1 expression via a Rho signalling pathway

**Figure 5:**
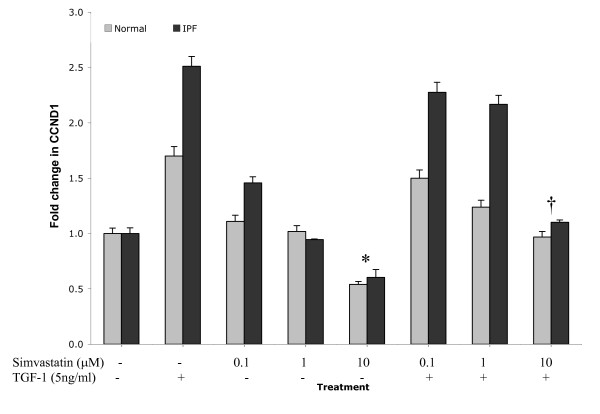
**Simvastatin abrogates cyclin D1 gene expression levels in a dose dependent fashion in human lung fibroblasts**. Serum deprived cells were incubated with Simvastatin (Sim 0.1–10 μM) for 16 hours. Subsequent TGF-β1 stimulation (5 ng/ml) was carried out for 8 hours. Experiments were performed in three separate human lung fibroblast cell lines from IPF patients (HIPF, LL29, and LL97a) and normal control equivalents CCD8LU. The control shown represents fibroblasts not exposed to Simvastatin and/or TGF-β1. The gene expression of cyclin D1 was then determined by real time PCR. Data shown demonstrates analysis from LL97a and CCD8LU fibroblasts. Data is representative of the mean of triplicate PCRs obtained from 3 independent experiments. Data are expressed as the mean fold change in cyclin D1 expression ± SEM. * = p < 0.05 compared to control untreated, † = p < 0.05 compared to TGF-β1 treatment.

The effect of cell pre-incubation with varying concentrations of Simvastatin (0.1 μM, 1 μM and 10 μM) on cyclin D1 gene expression in the IPF derived lung fibroblast cell lines and equivalent normal controls was analysed by real time PCR (Fig 5). The physiological concentrations of Simvastatin used abrogated cyclin D1 gene expression, irrespective of TGF-β1 presence (p < 0.05). Although 0.1 μM Simvastatin had little effect on cyclin D1 expression, 10 μM Simvastatin was efficacious enough to reduce even basal levels of cyclin D1 mRNA in test fibroblasts, inducing a 1.66 fold and 2.1 fold respective inhibition of the gene compared to untreated cells and TGF-β1-lone treated cells respectively. Furthermore the inhibition of cyclin D1 was further confirmed at the protein level by western blotting (data not shown). The data in fig 5 is from LL97a IPF derived lung fibroblasts; these data are also representative of the other 2 IPF lung fibroblast cell lines studied. Again trends were replicated within the normal fibroblast equivalents but with a lower magnitude of cyclin D1 expression compared to patient samples.

### Simvastatin induces G1 arrest in IPF lung fibroblasts

**Figure 6:**
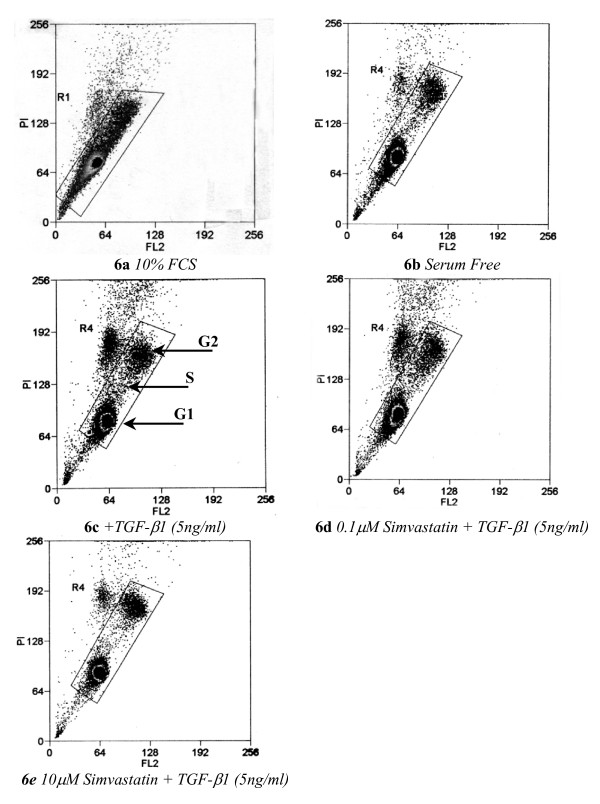
**Simvastatin influences cell cycle progression in human lung fibroblasts by inducing G1 arrest**. LL97a lung fibroblasts at 60% confluency were serum deprived for 48 hours; cells were then harvested following treatment (6a) DMEM containing 10% FCS (exponential growth) (6b) serum free (quiescent cells) (6c) TGF-β1 alone (5 ng/ml) (6d) Simvastatin (10 μM) alone (6e) Simvastatin (10 μM) and TGF-β1 (5 ng/ml) stimulation. FACS sorting was used to assess cell cycle progression using propidium iodide staining of cellular DNA content. Data are representative of FACS analysis performed in triplicate.

**Figure 7:**
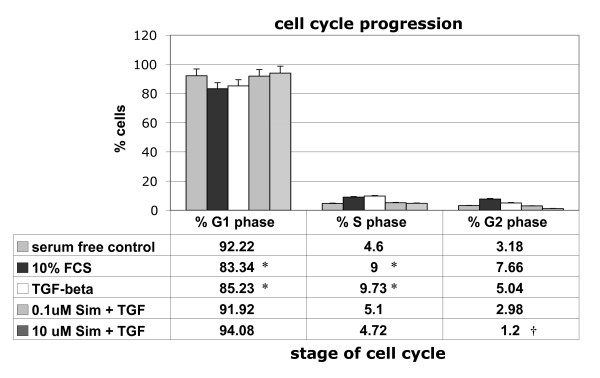
**Simvastatin modulation of cell cycle progression as determined by FACS analysis**. Data is summarised from the same treatments and FACS data from Fig 6 in LL97a lung fibroblasts. The % number of cells present in G1, S and G2 phase of the cell cycle are presented ± SEM and is representative of 3 independent experiments. * = p < 0.05 compared to serum free control, † = p < 0.05 compared to 10% FCS treatment.

To explore the influence, as yet unrecognised, of Simvastatin on IPF lung fibroblast proliferation, we analysed DNA content in Simvastatin-treated patient-derived lung fibroblasts (LL97a) using FACS analysis of propidium iodide stained of cells (Fig 6). Fibroblasts grown in DMEM containing 10% FCS (6a) showed their progression through the cell cycle; whereas serum deprivation limited G1 progression and entry in S phase by 51% (6b). Compared to serum-depleted samples, cells incubated in 5 ng/ml TGF-β1 (6c) presented a profile similar to that of cells grown in 10% FCS; with 5.04% of cells entering G1 phase and 9.73% of cells in S phase transition. Further analysis revealed that fibroblasts were G1 arrested following treatment with Simvastatin; small responses were observed at a dose of 0.1 μM (6d) and a more pronounced response is seen at the higher concentration of 10 μm (6e), irrespective of TGF-β1 treatment (5 ng/ml). Such cells were prevented from entering S phase of the cell cycle, thus reducing the percentage of cells in G2 phase of the cell cycle by 40.8% and 76.2% respectively. These findings are summarised in Fig 7 where Simvastatin is observed to induce a decrease in the percentage number of fibroblasts in G2 phase of the cell cycle with concurrent increase in cells remaining within G1 phase of the cell cycle.

## Discussion

Cyclin D1 is a critical regulator in progression of the cell cycle, specifically passage through the G1 phase and entry into S phase, beyond which cells are committed to mitosis. *CCND1 *is a recognised oncogene; thus, when *CCND1 *is over-expressed pathologically such as in oncogenesis, affected cells enter S phase more rapidly resulting in accelerated speed and frequency of proliferation [22]. There is increasing evidence that Rho family members promote cell cycle progression by regulating cyclin D1 and associated genes such as p21cip1, p27kip1 [23]. We have previously demonstrated that Rho is a key driver in fibroblast-mediated growth factor expression and myofibroblast formation [6,7]. In this study we have explored the role of cyclin D1 and interaction with RhoA signalling to determine key influences in observed fibroblast over-proliferation in IPF.

Our study data demonstrate for the first time that cyclin D1 gene and protein are upregulated in IPF-derived lung fibroblasts under basal proliferating conditions (media supplemented with 10% FCS). Indeed, levels of cyclin D1 mRNA expression greatly exceed those of the control cell line A431 that has a known 5-fold amplification of the gene [21]. The reason for the observed elevated levels of cyclin D1 in IPF cells lines is as yet unknown and will be addressed in separate lung tissue studies; however possibilities include amplification of gene copy number, hyper-stimulation of the RhoA pathway through an aberrant disease-associated mutation (*or *pathogenic mutation causing abrogation of pathway inhibitors) or simply, factor/s within the profibrogenic milieu. Nonetheless, the findings to date support our hypothesis that cyclin D1 deregulation could explain exaggerated fibroblast proliferation observed in IPF lungs, and possibly propagate, albeit partly, associated formation of fibroblastic foci. Interestingly, we observed that specific pro-fibrogenic growth factors, known to be associated with IPF pathogenesis [5], can induce cyclin D1 expression in serum-deprived fibroblasts. Cells treated with TGF-β1 show gene upregulation at both 1 ng/ml and 5 ng/ml, with the greatest response seen at the higher dose. CTGF at 10 ng/ml also induced cyclin D1 mRNA; however this trend was not replicated at the higher dose of 100 ng/ml in IPF fibroblasts. This result could be explained by CTGF-induced cell apoptosis in these cells at high concentrations [24].

We also believe that the growth factor effect on cyclin D1 expression in fibroblasts is not only dependent on the concentration of the particular mediator, but may also be factor-specific. Preliminary data in our laboratory reveals that another known pro-fibrogenic mediator, thrombin (1 ng/ml and 2.5 ng/ml) only induces small, insignificant responses in same fibroblast cyclin D1 expression. Thus not all fibrogenic growth factors have similar effects on *CCND1 *expression profiles; known differential effects of the test growth factors on the Rho signalling pathway may explain such discrepancy. Specifically, TGF-β1 and CTGF act via a Rho signalling pathway to induce changes in cyclin D1. However, thrombin has recently been shown to suppress RhoA activity by inducing tyrosine phosphorylation coinciding with a decrease in Rho activity [25]; accounting for its limited observed response on fibroblast cyclin D1 expression (in-house data).

Taken together, these observations support a crucial function for RhoA signalling in cyclin D1 expression in IPF lung fibroblasts, with consequence on their proliferative activity. We have demonstrated that inhibition of RhoA signalling (using both dominant negative transfection and pharmacological inhibitors) downregulates cyclin D1 expression in lung fibroblasts, reflected functionally, albeit indirectly, by altered cell turnover. There is evidence that there are 2 opposing mechanisms for Rho mediated control of cyclin D1; a stimulatory axis mediated through ERK signaling and a concurrent inhibitory axis acting through Rac/cdc42 [8]. These 2 mechanisms may account for some of the findings in this manuscript. We observe that constitutively active RhoA (G14V) augments cyclin D1 expression, however in separate experiments we also show that C3 exotoxin a Rho inhibitor is also able to increases cyclin D1 expression; thus suggesting that these 2 pathways may be active in the lung fibroblasts studied. Further experiments are needed to further identify the presence and role of ERK and Rac/cdc42 dependent pathways in relation to lung fibroblasts and IPF mechanisms. Also of interest is that the constitutively active RhoA construct (G14V) in the presence of TGFβ1 (5 ng/ml) is able to further elevate cyclin D1 mRNA expression in the IPF cell line with only little or no further effect in the control fibroblasts. Thus this may highlight a deregulated mechanism specific to the IPF cohort and thus present a suitable target for therapeutic intervention. We feel that this observation may be related to deregulation of pathways involved in suppression of cytokine signalling (SOCS) genes, which may increase IPF fibroblasts susceptibility to growth factors such as TGFβ1. This is a potential mechanism that has be highlighted in liver fibrosis [29] and emerging findings from our own experiments support the concept of deregulated SOCS 3 expression in IPF lung fibroblasts (in house data).

Experiments using the specific HMG CoA inhibitor agent, Simvastatin also support the concept that RhoA modulates cyclinD1 expression. Interestingly such statin agents possess increasingly recognised pleiotropic effects beyond that of cholesterol lowering, including CTGF inhibition, preventing myofibroblast formation and anti-fibrotic effects in kidney disease and heart disease [26,27]. These additional effects are due to Simvastatin's ability to modulate RhoA signalling; occurring as a result of inhibited post-translational modification of the RhoA molecule (a pre-requisite for its activation). Using Simvastatin we achieved abrogation of cyclin D1 mRNA and protein expression in a concentration dependent manner, irrespective of TGF-β1 conditioning. Simvastatin treatment was able to lower IPF fibroblast cyclin D1 levels to basal expression of normal cells. Functionally, Simvastatin also induced G1 arrest in the IPF fibroblasts, again overriding inductive effects of TGF-β1, resulting in suppressed cell proliferation. An alternative mechanism for the observed changes in cell cycle progression and cyclin D1 expression is Simvastatin-mediated disruption of lipid raft localisation. The lipid rafts are essential for efficient signal transduction by a number of cell types including B and T cells [28] resulting in altered growth factor and GTPase signalling such as Ras. However our data is consistent with Rho being the central mechanism for CCND1 disruption as the specific Rho inhibitor C3 exotoxin is able to influence expression, in addition we have preliminary data (in house data) in which we have utilised Simvastatin to inhibit GTPase activity, Rho activity can be restored by introducing geranylgeranylpyrophosphate (GGPP) with associated augmented cyclin D1 and growth factor expression. However restoring Ras activity by the incorporation of farnesylpyrophospahe (FPP) is unable to have the same effects and expression of cyclinD1 and other key growth factors is not returned. These observations may suggest that selective inhibitory manipulation of Rho signalling pathway components could be exploited to attempt therapeutic reversal of the fibroproliferative processes associated IPF.

## Conclusion

Our studies further enhance understanding of the pathogenic events within IPF lungs, highlighting fibroblast cell cycle deregulation via a cyclin D1 mechanism as a key factor in disease progression. Tentatively, we provide evidence to support future exploitation of direct RhoA inhibition (using HMG CoA inhibitor agents) as a novel strategic option for fibroproliferative abrogation in lung fibrosis.

## Abbreviations

α-Smooth Muscle Actin α-SMA

5-bromo-2'-deoxyuridine BrdU

cyclin D1 gene CCND1

Connective Tissue Growth Factor CTGF

Extracellular Matrix ECM

Fetal Calf Serum FCS

Fluorescence Activated Cell Sorting FACS

Farnesylpyrophosphate FPP

Geranylgeranylpyrophosphate GGPP

Glyceraldehyde-3-phosphate dehydrogenase GAPDH

Guanine nucleotide-binding regulatory protein G protein

Guanosine triphosphatase GTPase

3 hydroxy3methylglutaryl Coenzyme A HMG CoA

Idiopathic Pulmonary Fibrosis IPF

Phosphate buffered saline PBS

Reverse Transcription Polymerase Chain Reaction RT-PCR

Serum-free DMEM media SF-DMEM

Suppressor of cytokine Signalling SOCS

Transforming Growth Factor-β1 TGF-β1

## Competing interests

None of the authors are aware of any competing interests regarding submission/publication of this manuscript.

## Authors' contributions

*KW *has worked full time as a post-doctoral researcher on this project (funded by the British Lung Foundation) including its design, experimental work and data analysis; she has led production of this manuscript.

*EC *worked as a project student on the study under the guidance of KW and PH. EW helped perform the Simvastatin experiments and subsequent analysis that appears in Fig 5.

*PH *has given guidance to KW on experimental design and has helped in manuscript preparation.

*MS *is director of the lung fibrosis programme, closely supervising and advising KW; and has extensively revised manuscript drafts.
